# nc886, a non-coding RNA and suppressor of PKR, exerts an oncogenic function in thyroid cancer

**DOI:** 10.18632/oncotarget.11852

**Published:** 2016-09-06

**Authors:** Eun Kyung Lee, Seung-Hyun Hong, Sooyong Shin, Hyun-Sung Lee, Ju-Seog Lee, Eun Jung Park, Sun Shim Choi, Jae Woong Min, Daeyoon Park, Jung-Ah Hwang, Betty H. Johnson, Sung Ho Jeon, In-Hoo Kim, Yeon-Su Lee, Yong Sun Lee

**Affiliations:** ^1^ Center for Thyroid Cancer, National Cancer Center, Goyang, 410-769, Korea; ^2^ Cancer Genomics Branch, Research Institute, National Cancer Center, Goyang, 410-769, Korea; ^3^ Department of Biochemistry and Molecular Biology, University of Texas Medical Branch, Galveston, TX 77555, USA; ^4^ Department of Life Science and Center for Aging and Health Care, Hallym University, Chuncheon, 200-702, Korea; ^5^ Division of Thoracic Surgery, Michael E. DeBakey Department of Surgery, Baylor College of Medicine, Houston, TX 77030, USA; ^6^ Department of Systems Biology, University of Texas MD Anderson Cancer Center, Houston, TX 77030, USA; ^7^ Cancer Immunology Branch, National Cancer Center, Goyang, 410-769, Korea; ^8^ Department of Cancer System Science, Graduate School of Cancer Science and Policy, National Cancer Center, Goyang, 410-769, Korea; ^9^ Division of Biomedical Convergence, College of Biomedical Science, and Institute of Bioscience & Biotechnology, Kangwon National University, Chuncheon, 200–701, Korea

**Keywords:** nc886, thyroid cancer, protein kinase R, oncogene

## Abstract

nc886 is a recently identified cellular non-coding RNA and its depletion leads to acute cell death via PKR (Protein Kinase RNA-activated) activation. nc886 expression is increased in some malignancies, but silenced in others. However, the precise role of nc886/PKR is controversial: is it a tumor suppressor or an oncogene? In this study, we have clarified the role of nc886 in thyroid cancer by sequentially generating PKR knockout (KO) and PKR/nc886 double KO cell lines from Nthy-ori 3-1, a partially transformed thyroid cell line. Compared to the wildtype, PKR KO alone does not exhibit any significant phenotypic changes. However, nc886 KO cells are less proliferative, migratory, and invasive than their parental PKR KO cells. Importantly, the requirement of nc886 in tumor phenotypes is totally independent of PKR. In our microarray data, nc886 KO suppresses some genes whose elevated expression is associated with poor survival confirmed by data from total of 505 thyroid cancer patients in the Caner Genome Atlas project. Also, the nc886 expression level tends to be elevated and in more aggressively metastatic tumor specimens from thyroid cancer patients. In summary, we have discovered nc886's tumor-promoting role in thyroid cancer which has been concealed by the PKR-mediated acute cell death.

## INTRODUCTION

Thyroid cancer is the most common endocrine malignancy, whose worldwide prevalence has been soaring because of advances in medical imaging technologies and easy accessibility to ultrasonography [1, 2]. Since it remains asymptomatic even in advanced stages, many researchers have been seeking a biomarker to distinguish more aggressive tumors from indolent ones at the time of diagnosis. However, these efforts have not been successful.

Growing evidence indicates a significance for non-coding RNAs (ncRNAs) in the diagnosis and prognosis of thyroid cancer (reviewed in [3]). These ncRNAs are either small [< 50 nucleotides (nts)] or long (>200 nts) in length and play a regulatory role in gene expression by degrading target mRNAs or determining the chromatin status of target genes [4]. nc886 (a.k.a. pre-miR-886 or vtRNA2-1) is a recently identified regulatory ncRNA that is medium-sized (101 nts long) [5]. Not only in size but also in several other aspects, nc886 is distinct from those small or long ncRNAs. First, nc886 controls gene expression by binding and modulating the activity of a protein called PKR (*P*rotein *K*inase *R*NA-activated). Second, nc886 is transcribed by RNA polymerase III (Pol III) whereas a majority of regulatory ncRNAs are RNA polymerase II (Pol II) products.

nc886's role in cancer was first noticed because of its expression pattern in a number of cancer cell lines and patient samples [5–10]. Previous studies from our laboratory and others highlighted its putative tumor suppressor role, based on its epigenetic silencing in some malignancies. However, several lines of data indicate that the elevation of nc886 expression is a more general phenomenon, especially during early stages of tumorigenesis. The nc886 level is much higher in proliferating cells than non-proliferating tissues [5]. For example, most immortalized cells express nc886 abundantly ([5–8] and unpublished data from YSL). In many cases, this high level of nc886 is sustained or becomes even higher, as immortalized cells progress into more transformed cells. The elevated expression of nc886 in cancer is seen also in miRNA profiling data where probes (against miR-886-5p and -3p) detected nc886 [11–14]. All these observations coincide with higher Pol III activity in cancer cells than normal cells (reviewed in [15]).

nc886 plays a critical role in determining cell death or proliferation via its inhibitory function on PKR [6]. PKR was originally identified as a viral sensor that when activated upon viral infection led to cell death by phosphorylating eIF2α and consequently shutting down global cellular protein synthesis. In addition to viral sensing, PKR is involved in diverse cellular signaling pathways and is implicated in cancer. Although PKR's pro-apoptotic role initially suggested a tumor suppressor role, its precise function in cancer is still controversial (reviewed in [16]). In our published work [5, 6], epigenetic silencing of nc886 occurs in a subset of cancer cells (referred to as “nc886^−^ cells” hereafter) and nc886 knockdown (KD) activates PKR and consequently induces apoptosis, similarly to viral infection. This result led us to propose a “tumor sensing model” in comparison to PKR's original viral sensing role, to illustrate the significance of nc886/PKR in eliminating pre-cancerous cells [17].

Nevertheless, the precise role of nc886 in its relation with PKR in tumor promotion or suppression has yet to be elucidated. The elevated expression of nc886 in proliferating cells concurs with its oncogenic role which agrees with PKR's putative tumor suppressor function; however this idea has never been examined. Of course, nc886 may also play PKR-independent roles. Studies on nc886 have been hampered, because its depletion results in acute cell death via PKR activation. For this reason, it has been challenging to obtain an nc886^−^ cell line from its isogenic nc886^+^ control cell line. Comparison between the two cell lines is essential in order to evaluate nc886's precise role in cancer. In this study, we have solved this problem by sequentially generating PKR and nc886 knockout (KO) cells and, for the first time, clearly determined their roles in thyroid cancer.

## RESULTS

### Expression analysis of nc886 in patient samples and thyroid cell lines

**Figure 1:**
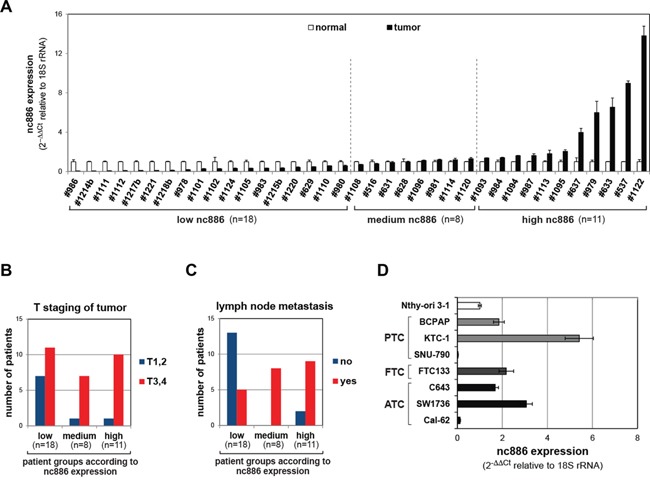
nc886 expression in tissue samples from thyroid cancer patients and cell lines **A.** qRT-PCR measurement of nc886 in 37 pairs of a thyroid tumor and its adjacent normal tissue. nc886 Ct values were normalized to 18S rRNA Ct values to calculate 2^−ΔΔCt^. Values of each tumor (black bars) are shown relative to its normal tissue whose values were set as 1 (plain bars). An average and the standard deviation from triplicate measurements are indicated. Patient identity is anonymously designated in #-number. Vertical dotted lines classify samples into low (tumor vs normal fc < 0.7), medium, and high (fc > 1.3) expression of nc886. The fc cutoffs were determined according to p-values (p < 0.05 in t-test) of the boundary samples. **B-C.** Sub-classification of patients in each nc886-expression group according to T staging (panel B) and lymph node metastasis (panel C). **D.** qRT-PCR measurement of nc886 in thyroid cell lines. The value of Nthy-ori 3-1 is set as 1. All other descriptions are the same as panel A.

We measured the expression of nc886 in paired normal/tumor samples from 37 thyroid cancer patients and classified into three groups (low, medium, and high) according to nc886 levels (Figure 1A). Elevated expression of nc886 in tumors was correlated with the number of primary tumors (p=0.028, Table 1), tumor aggressiveness (see T staging in Figure 1B, p for trend=0.050) and metastasis to lymph nodes (p=0.028, Figure 1C). However, there was no signficant association of nc886 with clinical variables such as the age at diagnosis, the size of primary tumors, and Hashimoto's thyroiditis, an autoimmune disease leading to chronic inflammation.

**Table 1:** Clinical characteristics of 37 patients with papillary thyroid cancer according to the level of nc886 in tumor specimen

	Total n=37	Expression level of nc886			P-value
		Low n=18	Medium n=8	High n=11	
Age at diagnosis (yrs)	47.5±11.2	50.2±10.8	42.0±10.2	47.1±12.0	0.228
Size of tumor (cm)	1.7±1.0	1.7±1.1	1.9±1.3	1.6±0.8	0.777
No of primary tumor	1.5±0.8	1.3±0.6	2.1±1.0	1.5±0.7	0.028
Extrathyroidal extension (n, %)	26(70.3%)	10(55.6%)	7 (87.5%)	9(81.8%)	0.157
Advanced T stage (T3-4)	28(75.7%)	11(61.1%)	7 (87.5%)	10(90.9%)	0.131^†^
Lymph node metastasis (n, %)	22(59.5%)	5(27.8%)	8 (100%)	9 (81.8%)	0.0005
N0	15(40.5%)	13(72.2%)	0(0%)	2(18.2%)	0.028
N1a	15(40.5%)	4(22.2%)	4(50.0%)	7(63.6%)	
N1b	7 (18.9%)	1 (5.6%)	4(50.0%)	2 (18.2%)	
Hashimoto's thyroiditis	16(43.2%)	6 (33.3%)	4 (50.5%)	6(54.5%)	0.486

* mean ± standard deviation

† P for trend = 0.050

We also measured nc886 expression in thyroid cell lines and found it to be higher in the majority of cancer cell lines than in an immortalized cell line Nthy-ori 3-1 (Figure 1D). Nthy-ori 3-1 cells, although not fully-transformed, proliferated well and expressed nc886 much more abundantly than non-proliferating thyroid tissues (Supplementary Figure S1A). Very low expression of nc886 was also previously seen in normal tissues from several organs [5], suggesting that nc886 expression is proportional to cell proliferation rates. Actually, nc886 expression became less when cell proliferation was slowed by reducing the serum concentration in the culture medium (Supplementary Figure S1B).

The above nc886 expression pattern, together with its association with cell proliferation *in vitro* as well as tumor progression and aggressiveness in patients, supported a putative oncogenic role in thyroid cancer. However, nc886 was epigenetically silenced in a subset of thyroid cells (Supplementary Figure S2A-B), as previously seen in other types of cancer including esophageal squamous cell carcinoma, gastric cancer, acute myeloid leukemia, and lung cancer [7–10]. Therefore we could not rule out the possibility of a tumor suppressor role. To clarify the role of nc886 in thyroid cancer, we attempted to assess its loss-of-function phenotypes.

### Acute cell death triggered by nc886 silencing and the consequent PKR activation

**Figure 2:**
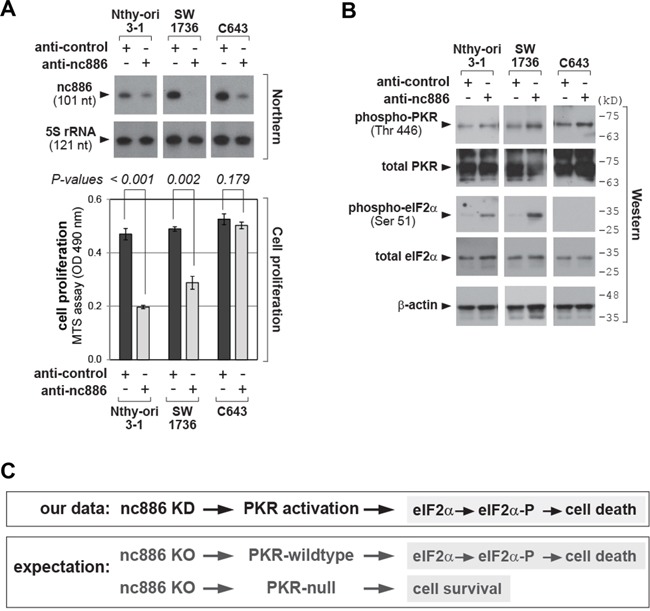
nc886 KD activates PKR, which impairs cell proliferation **A.** Northern hybridization of nc886 and 5S rRNA as a loading control (top panel) and cell proliferation (MTS) assays (bottom panel) after nc886 KD. “anti-nc886” (an anti-oligo targeting nc886) and “anti-control” (targeting a paralog ncRNA vtRNA1-1 but not nc886) were transfected into indicated cells at 100 nM for 48 hrs. **B.** Western blot of indicated proteins after nc886 KD in panel A. Molecular sizes in kilodalton (kD) from the size marker are indicated on the right. **C.** Summary of nc886 KD data from panel A-B and expected cellular outcomes upon nc886 KO.

As stated in the Introduction, nc886 is a repressor of PKR. When we transfected an antisense oligonucleotides (anti-oligo) targeting nc886 into Nthy-ori 3-1, SW1736, and C643 thyroid cell lines, nc886 expression level was diminished as shown by our Northern blot in Figure 2A. nc886 KD led to PKR activation, as indicated by the increase of phospho-PKR which is the active form (Figure 2B). The active PKR phosphorylated its best substrate eIF2α and consequently inhibited cell proliferation in the immortalized Nthy-ori 3-1 line as well as in a thyroid cancer cell line SW1736 (Figure 2A–2B). In contrast, neither eIF2α phosphorylation nor an effect on cell growth was observed in the other cancer cell line C643.

At first glance, this impaired cell proliferation upon nc886 KD seemed to agree with its putative oncogenic role. However, this phenomenon should be understood as the PKR-dependent “tumor sensing model” (see Introduction; [17]) rather than nc886's role in the etiology and/or progression of thyroid cancer. In other words, nc886 KD immediately provoked the PKR cell death pathway before we were able to observe any other phenotypes that could truly reflect the functional significance of nc886's elevated expression in immortalized or transformed cells. To elucidate this, it would be essential to examine long-term cellular phenotypes and make a comparison between nc886-null (nc886^−^) cells and isogenic nc886^+^ cells. Thus, nc886 KO cell lines were generated.

### Sequential generation of PKR and PKR/nc886 double KO cell lines

Since anti-oligos do not self-propagate and thus are inappropriate for long-term KD, we took advantage of a new gene-editing technique adapted from a bacterial immune system composed of “Clustered Regularly Interspaced Short Palindromic Repeats (CRISPR)” and “CRISPR-associated genes (Cas)” (reviewed in [18]). PKR also posed a problem when generating nc886 KO cells because those cells (nc886^−^) are expected to die in the presence of PKR (Figure 2C). C643 cells could be a choice because they were resistant to PKR-mediated cell death (Figure 2A–2B). However, this resistance indicated that the PKR pathway had already gone awry. So it would be questionable whether any data from this cell line would appropriately reflect the role of nc886, in concert with PKR, in thyroid tumorigenesis.

Our maneuver to solve this situation was to make PKR KO cell lines ahead of nc886 KO. In this way, comparison among wild type (designated as PKR^wt^/nc886^wt^), PKR KO (PKR^KO^/nc886^wt^), and double KO (PKR^KO^/nc886^KO^) will answer to the question about the precise contribution of nc886 and/or PKR in thyroid tumorigenesis. We chose Nthy-ori 3-1, because it is not a fully transformed cell line and so is likely to retain a more natural PKR pathway as compared to cancer cell lines. For the same reason, Nthy-ori 3-1 cells could transform in either direction: more tumorigenic or less upon nc886/PKR KO. This would be a considerable advantage because nc886 and PKR KO were expected to show opposite phenotypes.

We designed a small guide RNA (sgRNA) targeting amino acid #15-21 in the first exon of the PKR gene (Supplementary Figure S3A). We selected PKR^KO^ clones that were certified to have frameshifting 1-2 nt deletions by DNA sequencing (Supplementary Figure S3B-C) and accordingly no PKR protein expression by Western blot (Supplementary Figure S4A-B).

**Figure 3:**
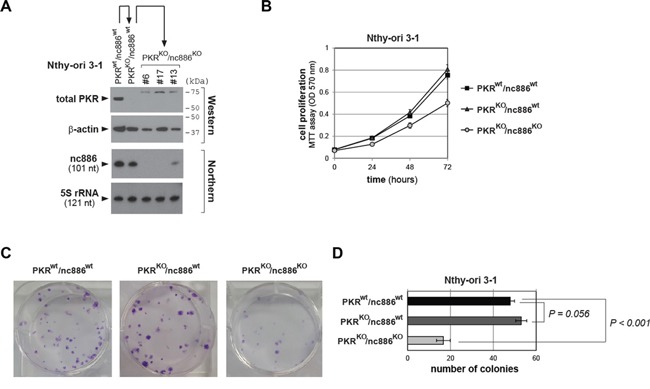
Cell proliferation assays of PKR or nc886 KO cells **A.** Confirmation of KO cell lines by Western/Northern blots of PKR/nc886, with β-actin and 5S rRNA as loading controls respectively. Protein size markers are indicated on the right. Among three PKR^KO^/nc886^KO^ clones shown here, functional assay data from clone #6 are shown in main figures and data from #17 and #13 shown in Supplementary Figures. **B.** MTT cell proliferation assays of indicated KO lines. An average and the standard deviation from nonaplicate (n=9; for PKR^wt^/nc886^wt^ and PKR^KO^/nc886^wt^) or triplicate (n=3; for PKR^KO^/nc886^KO^) experiments are plotted and shown. **C-D.** Colony formation assays. A representative plate from each cell line after crystal violet staining is shown in panel C. In panel D, colony numbers were counted independently from nine plates (for PKR^wt^/nc886^wt^ and PKR^KO^/nc886^wt^) or three plates (for PKR^KO^/nc886^KO^). An average and the standard deviation are calculated and shown.

Next we attempted to generate nc886^KO^ from PKR^KO^ clones. Because nc886 is an ncRNA, a deletion of 1-2 nts does not guarantee that it will be a functional KO. Therefore, we designed two sgRNAs (yellow highlighted in Supplementary Figure S5A) flanking the nc886 transcript (blue letters in Supplementary Figure S5A) in order to remove the whole DNA segment between them. These two sgRNA-expressing plasmids were transfected together into parental Nthy-ori 3-1(PKR^wt^) and Nthy-ori 3-1(PKR^KO^) cells. During initial selection of nc886^KO^ candidate clones, we noticed that colonies were barely recovered from the original (PKR^wt^) Nthy-ori 3-1 cells whereas a decent number of colonies were recovered from all four PKR^KO^ clones (Supplementary Figure S6). This result corroborated our idea that nc886 depletion is deleterious to cell proliferation and PKR activation is certainly one cause for it. We have obtained several PKR^KO^/nc886^KO^ subclones which were confirmed by the appearance of a shorter PCR product indicative of a deletion of the nc886 DNA segment between the two sgRNAs (Supplementary Figure S5B) as well as by sequencing (a representative result from nc886^KO^ clone #6 shown in Supplementary Figure S5C). We also ascertained no nc886 expression by Northern hybridization (Figure 3A) in three PKR^KO^/nc886^KO^ clones (#6, #13, and #17 in Figure 3A) and used these cell lines to examine nc886's role in thyroid cancer.

### Retardation in cell growth, migration, and invasion in nc886^KO^ cells

**Figure 4:**
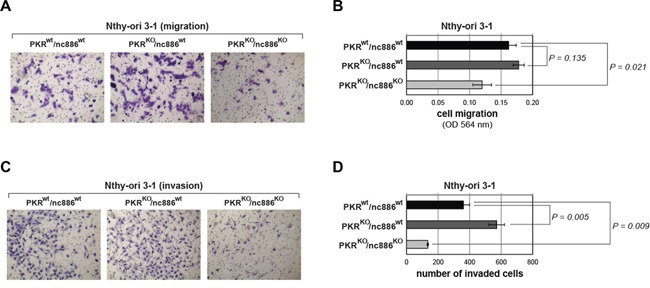
Cell migration and invasion assays for PKR or nc886 KO cells Representative images after the assays **panels A** and **C.** and their quantification **panels B** and **D.** are shown. All the other descriptions (for example, the number of replicates for each cell line) are the same as Figure 3B-D.

Our MTT and colony formation assays demonstrated that PKR^KO^/nc886^KO^ cells grew more slowly than nc886^wt^ cells (PKR^wt^/nc886^wt^ and PKR^KO^/nc886^wt^ in Figure 3B–3D and Supplementary Figure S7A-C). PKR KO itself conferred a slight growth advantage, as could be seen by the larger colony sizes of PKR^KO^/nc886^wt^ cells than those of PKR^wt^/nc886^wt^. However, MTT values and colony numbers were not so significantly increased in the absence of PKR (Figure 3B–3D and Supplementary Figure S7A-C). Cell migration and invasion assays yielded similar results. PKR^KO^/nc886^KO^ cells were clearly less migrating and invasive than PKR^KO^/nc886^wt^ cells; PKR^KO^/nc886^wt^ cells were slightly more than PKR^wt^/nc886^wt^ cells (Figure 4A–4D and Supplementary Figure S8A-D). Collectively from all these data, it was evident that nc886 played an oncogenic role.

It should be noted that nc886^KO^ cells were in the PKR^KO^-background and therefore all the phenotypes manifested by nc886 KO were PKR-independent. For example, nc886's pro-proliferative role cannot be attributed to its inhibition of PKR's pro-apoptotic function. If PKR inhibition were nc886's sole function, PKR^KO^/nc886^KO^ and PKR^KO^/nc886^wt^ cells would have shared an identical phenotype. Another important conclusion from our data is the contribution of PKR, which when alone appeared to play a marginal role.

### Gene expression profiling in PKR^KO^ and nc886^KO^ cells

**Figure 5:**
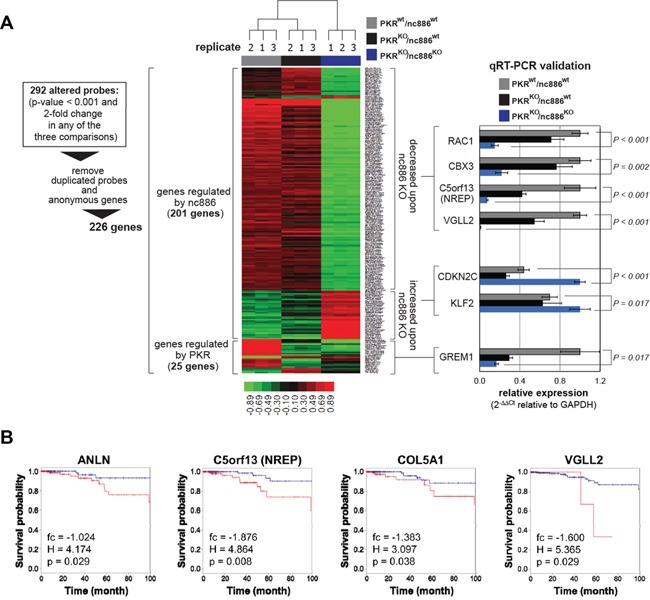
Comparison of gene expression profiles among PKR^wt^/nc886^wt^, PKR^KO^/nc886^wt^, and PKR^KO^/nc886^KO^ cells **A.** A heat map showing hierarchical clustering of 226 genes whose expression values were significantly (log2(fc) > 1 or < −1) altered in PKR or nc886 KO. The complete list of 226 genes is shown in Supplementary Table S1. 201 and 25 genes were altered upon nc886 and PKR KO respectively. Some genes were chosen for qRT-PCR measurement as described in Figure 1A except that GAPDH was used for normalization. **B.** Kaplan-Meier curves stratifying survival of 505 patients (the TCGA cohort) according to the expression of indicated genes. In each graph “fc”, “H”, and “p” values are displayed. “fc” is the expression value (in a log2-transformed scale) of indicated genes in PKR^KO^/nc886^KO^ relative to PKR^KO^/nc886^wt^ in this array data. “H” and “p” indicate hazard ratio of cox regression and p-value of cox regression respectively.

Next we further investigated the molecular basis of nc886/PKR's role. We performed microarray experiments in the three cell lines (PKR^wt^/nc886^wt^, PKR^KO^/nc886^wt^, and PKR^KO^/nc886^KO^) and obtained a list of 226 genes from expression changes between them (as diagrammed in Figure 5A; Supplementary Table S1 for the full list). As shown in a heatmap (Figure 5A, left panel), expression of 25 and 201 genes were altered respectively by PKR and nc886 KO. The microarray data were validated by measuring some altered genes by qRT-PCR (Figure 5A, right panel).

We mainly focused on the 201 genes for further analysis. Gene ontology (GO) analysis revealed that 13 GO sets were altered significantly (p<0.05) upon nc886 KO and most of them were implicated directly or indirectly in tumorigenesis (Table 2). Among them, regulation of cell cycle, cell growth, and apoptosis as well as cytoskeleton organization (bold-highlighted GO terms in Table 2) might explain the nc886 KO phenotypes in cell proliferation, migration, and invasion (Figure 3–Figure 4). For example, the expression of anti-apoptotic proteins, such as EGFR, HIPK2, HSPBL2 and TAX1BP1 (see “Negative regulation of apoptosis” in Table 2), was decreased upon nc886 KO (Supplementary Table S1) and this may explain the diminished growth of the PKR^KO^/nc886^KO^ cells.

**Table 2:** Gene Ontology analysis of the 201 nc886-regulated genes

GO	p-value	Genes
Regeneration	0.006	INA, CDKN1A, CCL2, MAP1B, VCAN
Regulation of transforming growth factor beta receptor signaling pathway	0.007	CDKN1C, HTRA1, HIPK2, C5ORF13
Localization within membrane	0.010	EGFR, RAC1, MAL
Cellular response to stress	0.014	DBNL, CCM2, NUDT1, MAP1B, PMS2L4, RPA3, CDKN1A, EYA2, MAP1LC3A, POLD2, INSIG1, HIPK2, VACN
**Cytoskeleton organization**	0.015	ABLIM1, INA, LIMA1, STMN3, TMSB15A, MAP1B, RAC1, RALA, ANLN, CNN1, KRT86
**Cell death**	0.016	GGCT, AIMP2, HSPBL2, GARS, TBRG4, RNF216, OPTN, TAX1BP1, DDIT4, EYA2, F3, HIPK2, RAC1, TGM2, SRGN
**Regulation of cyclin-dependent protein kinase activity**	0.018	EGFR, CDKN1C, CDKN1A, CDKN2C
Negative regulation of BMP signaling pathway	0.019	BMPER, HTRA1, HIPK2
**Interphase of mitotic cell cycle**	0.022	EGFR, CDKN1C, CDKN1A, CDKN2C, TBRG4
**Negative regulation of apoptosis**	0.030	EGFR, CDKN1A, CCL2, SFRP1, F3, HIPK2, HSPBL2, TGM2, TAX1BP1
Vascularture development	0.045	CCM2, PDPN, CTGF, EFNB2, TGM2, SOX18, COL5A1
Response to wounding	0.047	INA, CCL2, PDPN, CTGF, F3, MAP1B, RAC1, VCAN, AFAP1L2, CTSB, COL5A
**Regulation of cell growth**	0.050	CDKN1A, CDKN2C, CTGF, HTRA1, MAP1B, NPPB

Significantly (p<0.05) enriched GO terms and names of individual genes therein

We also performed a gene-network analysis using the GeneMANIA plug-in tool in Cytoscape (http://www.cytoscape.org/; [19]) to identify the direct physical interactions and interconnection of pathways among the 201 genes. In this network (Supplementary Figure S9), p53 and MYC were prominent hubs. p53 and MYC are the most renowned tumor suppressor and oncogene respectively and, interestingly, are known to modulate Pol III transcription and therefore nc886 expression ([15] and our unpublished data). Further studies will be needed to unravel the relation of nc886 to p53, MYC, and the 201 genes.

Next we sought to examine data from The Cancer Genome Atlas (TCGA) to interrogate further nc886's clinical significance. The TCGA does not contain expression data of nc886 itself, because nc886 belongs to neither mRNA nor miRNA and therefore was excluded from most array or sequencing platforms. Instead, we examined expression of the 201 nc886-regulated genes in the TCGA RNA-seq data from 505 thyroid cancer patients (Supplementary Figure S10) to analyze their implication in patient survival. We observed that lower expression of ANLN, C5orf13 (= NREP), COL5A1, VGLL2, C15orf52, and KIAA1644 was significantly (p<0.05) associated with longer survival of the patients (Figure 5B and Supplementary Figure S11), in agreement with their lowered expression levels upon nc886 KO.

The array data were also analyzed against the molecular signature database (MSigDB; http://software.broadinstitute.org/gsea/index.jsp) to see which transcription factor (TF) target genes were enriched or depleted (Supplementary Figure S12A and Supplementary Table S2). We could not find any consistent elevation or depletion of TF targets upon nc886 KO. This was not so surprising because nc886 is exclusively localized to the cellular cytoplasm [5] and so any direct effect on TFs would be unlikely. Only one exception was the serum response factor (SRF) signature which was enriched upon nc886 KO (Supplementary Table S2). It needs to be clarified whether the increased activity of SRF was a direct consequence of nc886 KO.

Although PKR KO exhibited marginal phenotypes (Figure 3–Figure 4) and also only a moderate change in gene expression (Figure 5A), we found a number of TF target genes to be altered significantly (Z-score cutoff = 3; Supplementary Figure S12A). Nine top altered TFs (Z-score cutoff = 4) were all depleted in PKR^KO^ cells and notably seven were the NF-κB TF (dark blue bars in Supplementary Figure S12B). In addition, our pathway analysis using Biocarta identified one enriched pathway and 13 depleted pathways (Z-score cutoff = 4; Supplementary Figure S12C) among which seven were implicated in NF-κB (dark blue bars in Supplementary Figure S12C). This result corroborated the long-standing idea that PKR is the upstream of NF-κB.

## DISCUSSION

**Figure 6:**
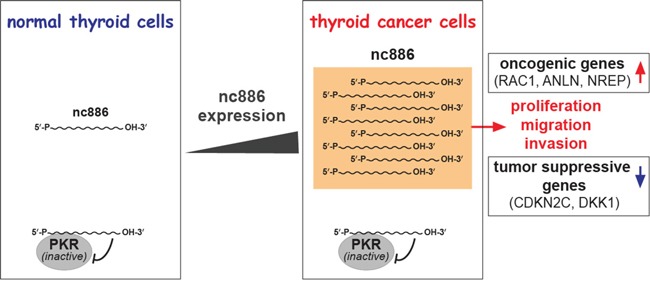
Summary cartoon for nc886's role in thyroid cancer

In this study, we have clearly determined nc886's role in thyroid cancer using CRISPR/Cas-mediated gene KO and provided a novel finding that nc886 plays a putative oncogenic role (summarized in Figure 6). nc886 expression is elevated in clinical samples. nc886 KO led to decreased tumor phenotypes and also to altered expression of a set of genes, some of which have been identified as supportive of nc886's putative oncogenic roles. For example, nc886^KO^ cells had elevated expression of CDKN2C and DKK1, known to inhibit thyroid cell proliferation and migration when overexpressed [20, 21]. However, the expression of ANLN, and C5orf13 (a.k.a. NREP and P311) was decreased in nc886 KO (Figure 5). These genes have been reported to inhibit tumor cell proliferation and migration when suppressed [22, 23] and their lower expression was associated with good survival of patients in the TCGA cohort (Figure 5). Therefore, nc886 and some of its associated genes could be an informative marker in deciding the extent of surgery in preoperative settings and in determining the dose of radioactive iodine therapy after surgery to improve the patient's outcome.

We also clarified PKR's role in thyroid tumorigenesis in this study. To the best of our knowledge, our report is the first to utilize PKR KO human cells to evaluate tumor phenotypes. From our data in PKR^KO^ cells, PKR *per se* plays an insignificant role or only a very slight tumor suppressor role. Our results here correspond with several lines of mouse data having shown no evident tumor phenotypes from PKR-null mice [24] or having indicated a moderate tumor suppressor role in certain experimental conditions [25, 26]. We surmise that PKR, only when activated, can play dual roles in cancer: inducing apoptosis to eliminate pre-cancerous cells or triggering pro-inflammatory responses. Apoptosis affects tumorigenesis negatively but a pro-inflammatory response affects a malignancy positively. In either case, PKR is not a direct driver or suppressor in tumorigenesis. Rather, we infer that the activation of PKR serves as a sentinel or a facilitator in cancer. In this regard, it would be informative to consider nc886's role in relation with PKR in thyroid cancer. Our results here showed that nc886 silencing occurred in some thyroid cancer cells (Supplementary Figure S2) as reported in other types of cancer [7–10] and that nc886 KD activated PKR (Figure 2). More efforts will be needed to elucidate the contribution of nc886/PKR to thyroid tumorigenesis.

In summary, we have uncovered nc886's putative oncogenic role in thyroid cancer (Figure 6). Previous studies have highlighted nc886's putative tumor suppressor role mainly because its epigenetic silencing was more easily recognized in cancer cells. We presume that nc886's role in cancer is multifaceted and that its precise function will be determined by several factors such as cancer types, stages, and cellular contexts. Since it is clear from our data that nc886's oncogenic function is independent of PKR, we are currently seeking to identify its regulatory target molecule(s) in order to elucidate definitely the molecular mechanisms of malignancy.

## MATERIALS AND METHODS

### Cell lines and tissue samples

The following cell lines were used in this study: Nthy-ori 3-1, an SV-40 immortalized cell line derived from normal primary thyroid follicular epithelial cells [27]; BCPAP, KTC-1, SNU-790 from papillary thyroid carcinoma; FTC133 from follicular thyroid carcinoma; C643, SW1736, and Cal-62 from anaplastic thyroid carcinoma (for SNU-790, [28]; for all other cell lines, [29] and references therein). Nthy-ori 3-1 was purchased from Sigma-Aldrich (St. Louis, MO, USA); BCPAP and Cal-62 were from DSMZ (Deutsche Sammlung von Mikroorganismen und Zellkulturen, Germany); C643 and SW1736 were from CLS (Cell Line Service, Germany); and KTC-1, SNU-790 and FTC133 lines are our laboratory stocks (National Cancer Center, Center for Thyroid Cancer, Korea). Cells were cultured in appropriate growth media supplemented with 10% fetal bovine serum (FBS) (GE Healthcare Life Sciences; Logan, UT, USA) and 1% antibiotic-antimyotic (Life Technologies; Carlsbad, CA, USA). All cell lines were grown in a humidified incubator in 5% CO_2_ at 37°C.

Normal and tumor tissues were obtained from thyroid cancer patients who underwent thyroid surgery at the National Cancer Center (NCC) Hospital. All fresh tissues were frozen immediately after surgery in liquid nitrogen and stored at −70°C according to the protocols approved by the institutional review board, NCC for the human subject guideline of NCC (NCC2014-0003) that is in accordance with the principles of the Declaration of Helsinki. Hospital medical records and pathology reports of 37 patients who underwent surgery and diagnosed as papillary thyroid cancer in our clinic were reviewed. Pathologic staging was defined according to the TNM classification system of International Union against Cancer and the American Joint Committee on Cancer, 7^th^ edition. The clinical and pathological features of the patient are summarized in Table 1.

### Transfection, anti-oligos, RNA/protein isolation and measurement

The source of general reagents was described in [5, 6, 8]. Anti-oligos (“anti886 75-56” and “anti-vt 21-2” which are designated as “anti-nc886” and “anti-control” respectively in this work) were prepared and transfected as described in [5]. Total RNA from patient tissue samples and cell lines was isolated by Trizol reagent (Life Technologies; Carlsbad, CA, USA). Northern hybridization, qRT-PCR measurement of nc886 and control genes, Western blot of PKR and other proteins were performed as previously described [5]. Sequence information on qRT-PCR primers is available upon request.

### Generation of CRISPR/Cas KO cell lines

“hCas9” and “gRNA_Cloning Vector” were purchased from Addgene (plasmid #41815 and #41824 respectively). A sgRNA-expressing plasmid for PKR (“pCR sgPKR-1a”) was a kind gift from Dr. Stacy Horner at Duke University. sgRNA-expressing plasmids for nc886 were constructed according to the gDNA synthesis protocol (https://www.addgene.org/41824/ and [30]). Briefly, inserts were made by annealing two partially complementary oligos containing sgRNA sequences (shown in Supplementary Figure S5A) and converting into a fully double-stranded DNA fragment by Phusion™ High-Fidelity DNA Polymerase (New England Biolabs; Ipswich, MA, USA). The inserts were incorporated into *Afl*II-linearized “gRNA_Cloning Vector” by using the Gibson assembly kit (New England Biolabs), to yield “pCR sg886-164” and “pCR sg886+15”.

The Cas9-expressing plasmid (“hCas9”), in combination with “pCR sgPKR-1a” (for PKR KO) or with “pCR sg886-164” and “pCR sg886+15” (for nc886 KO), was transfected by using Lipofectamine 2000 (Life Technologies). Untransfected cells were processed in parallel as a negative control during G418 selection. At 24 hrs after transfection, cells were transferred to a growth medium containing 1 mg/ml of G418. G418-resistant colonies were individually isolated and further cultured.

### Cell proliferation, migration, and invasion assays

Cell proliferation measurement was based on the conversion of 3-(4,5-dimethylthiazol-2-yl)-2,5-diphenyl tetrazolium bromide (MTT) dye or its derivative MTS dye. MTT and MTS dyes were purchased respectively from Sigma-Aldrich and Promega (Madison, WI, USA) and assays were performed according to the manufacturer's instructions. In colony formation assays, 100 cells were seeded into one well of a 6-well plate and maintained for 7 days. Colonies were fixed and stained with 1% crystal violet to count colony numbers.

Cell migration assays were performed using 8-μm pore filter insert (BD Biosciences; San Jose, CA, USA). Cells were resuspended in a serum-free RPMI-1640 medium, were added onto the upper chamber, and were allowed to migrate to the lower chamber containing RPMI-1640 with 1% FBS for 24 hrs. Migrated cells on the bottom surface of the insert were fixed, air-dried for 20 min, and stained with 1% crystal violet for 20 min. Remaining cells on the top surface were removed by wiping with a cotton swab. To quantify migration rates, inserts were placed in 10% acetic acid and the absorbance at 564 nm was measured. Cell invasion assays were conducted with matrigel-coated insert (BD Biosciences). Cells were stained with Diff-Quik stain™ (Sysmex; Kobe, Japan) and counted.

### mRNA microarray and pathway analysis

mRNA expression profiling and analysis of molecular signatures were performed as described in [7, 8]. Hierarchical clustering and generation of a heat map were done by using Cluster 3.0 and Java TreeView (version 1.1.6r4) softwares. Direct physical interactions and their corresponding pathways were estimated using the “GeneMANIA” Cytoscape plug-in function (http://www.cytoscape.org/) [19].

### TCGA data analysis

RNA-seq data and clinical data from 505 thyroid cancer patients were downloaded from the TCGA database (https://tcga-data.nci.nih.gov/tcga/tcgaHome2.jsp). A total of 20,132 genes available in the TCGA RNA-seq data were collected for further analysis. TCGA provides RNA-seq data in Expectation-Minimization values (RSEM) as normalized expression levels. Expression fold-change (fc) of a gene was estimated by dividing the RSEM of a gene by the average of RSEM estimated from the total 505 patient samples and was log2-transformed. Following the procedure previously reported [31], RSEM values of 0 ~ 0.1 were all replaced to 0.1 to avoid the infinity problem in calculating the expression fc. According to expression levels of a selected gene, the patients were classified into “high-expression group” (if a log2(fc) value is > 0)” and “low-expresion group” (< 0), as described previously [32]. Among 201 nc886-regulated genes (Figure 5), 189 genes were found in the gene list of the TCGA RNA-seq data and were used for survival analysis (see Supplementary Figure S10). The R studio was used for all statistical tests and analyses (http://www.rstudio.com/). The ‘survival’ packages were used (http://r-forge.r-project.org) to draw Kaplan-Meier curve and to calculate the Cox proportional hazard ratio.

### Statistical analysis

Descriptive analyses were performed to identify patient demographics. For analysis of categorical variables, frequencies and percentages were calculated. The patients were divided into three groups (low, medium, and high) according to the expression of nc886 in thyroid cancer tissues. Proportions in each group were compared using the χ^2^ and Fisher's exact test. For analysis of continuous variables, means and standard deviations were determined. The differences of the continuous variables were analyzed using Mann-Whitney *U* test, Student's unpaired two-sided *t*-test or a one-way ANOVA. Statistical analyses were performed with STATA software (version 10, StataCorp., College Station, TX, USA). All p-values were two-sided, and p-values less than 0.05 were considered statistically significant.

## SUPPLEMENTARY FIGURES AND TABLES






