# Supplementary material for: nc886, a non-coding RNA and suppressor of PKR, exerts an oncogenic function in thyroid cancer
Source: Oncotarget. 2016 Sep 6;7(46):75000–12. doi: 10.18632/oncotarget.11852 (PMC5342718; doi:10.18632/oncotarget.11852)
Supplement: Supplementary file 1 [file oncotarget-07-75000-s001.pdf]

nc886, a non-coding RNA and suppressor of PKR, exerts an oncogenic function in thyroid cancer

SUPPLEMENTARY FIGURES AND TABLES

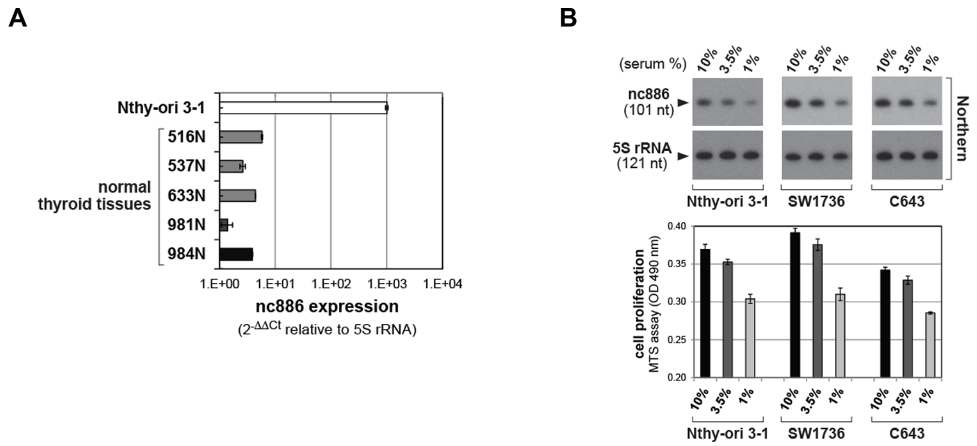

Supplementary Figure S1: nc886 expression is positively correlated to the degree of cell proliferation. A. qRT-PCR measurement of nc886 in Nthy-ori 3-1 and normal thyroid tissues. All other descriptions are the same as Figure 1, except that 5S rRNA was used for normalization. **B.** Northern hybridization of nc886 and 5S rRNA as a loading control (top panel) and cell proliferation (MTS) assays (bottom panel). Cells were cultured in a growth medium containing indicated concentrations of FBS for 72 hrs and then assays were performed.

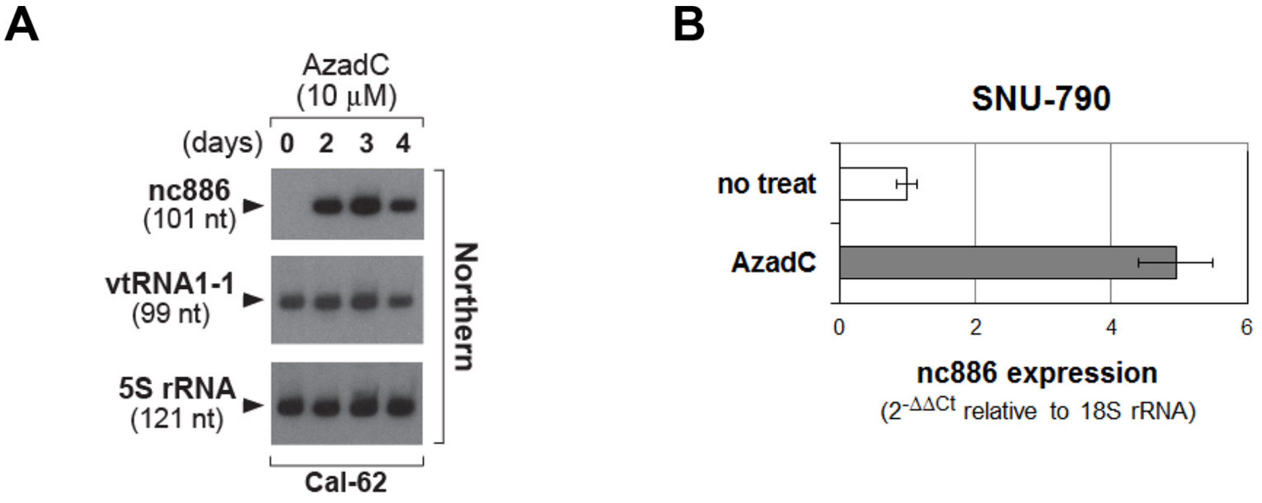

Supplementary Figure S2: Silencing of nc886 in Cal-62 and SNU-790 cells is due to CpG DNA hypermethylation. A. Northern hybridization of nc886 expression. Cal-62 cells were treated with 10 µM of 5-Aza-2'deoxycytidine (AzadC) which is an inhibitor of CpG DNA methylatransferase for indicated days. vtRNA1-1, a ncRNA that is paralogous to nc886 but is distinct from it, is shown for comparison. 5S rRNA is for equal loading of RNA. **B.** qRT-PCR measurement of nc886 after SNU-790 cells were treated with 10 µM AzadC for three days. All other descriptions are the same as panel A and Figure 1A.

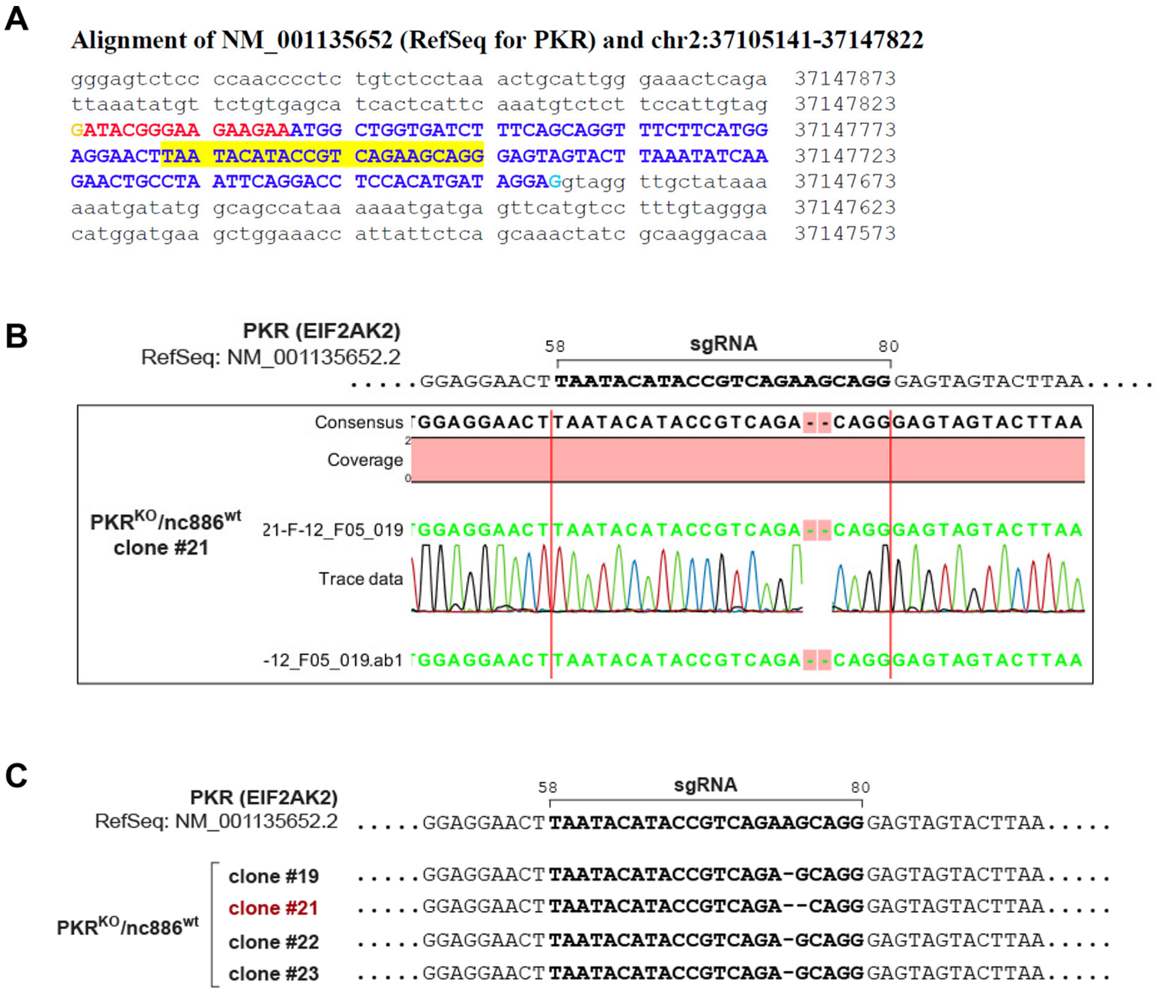

Supplementary Figure S3: CRISPR/Cas KO of PKR. A. Screenshot of PKR mRNA/genomic alignment (from the UCSC genome browser) to show the sgRNA sequence (yellow-highlighted) used for PKR CRISPR/Cas in this study. Small and capital letters designate introns and the first exon respectively. Red and blue capital letters are the 5'-untranslated region and open reading frame respectively. The numbers on the right are genomic coordinates. **B-C.** Alignment of our sequencing data from candidate PKR^{KO} clones to the reference sequence (RefSeq). A representative sequencing chromatogram is shown in panel B. Four clones have 1-2 nts deletion at the sgRNA region and thus have frameshifting mutations.

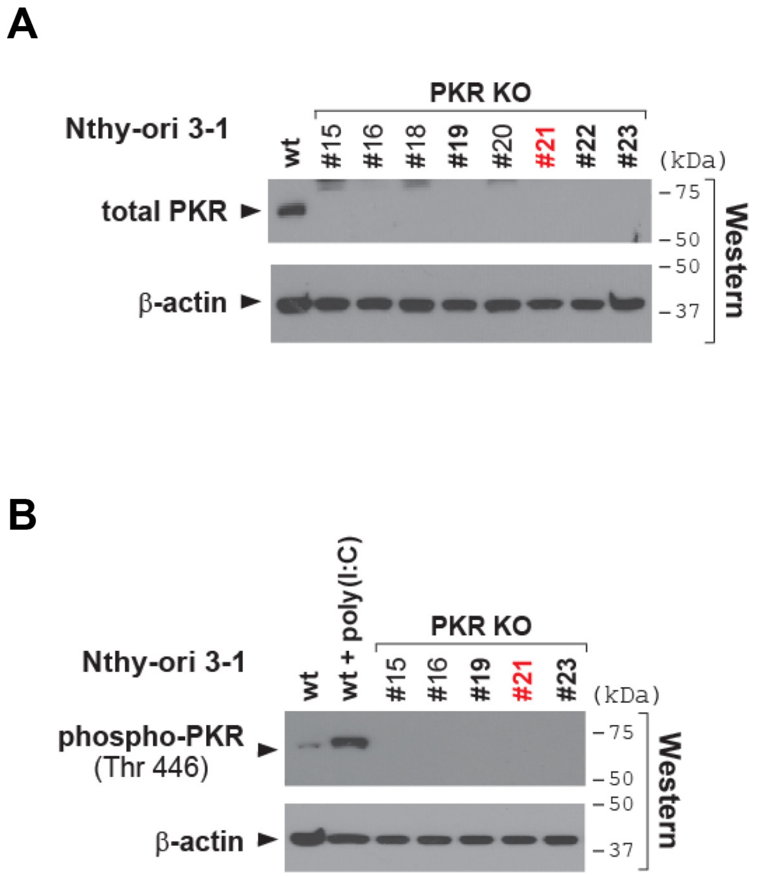

Supplementary Figure S4: Validation of PKR^{KO} candidate clones by Western blot. Western blot of total PKR **panel A.** and phospho-PKR **panel B.** together with β-actin as a loading control. The size markers are indicated on the right. In panel B, to ascertain the band identity, cells were treated with synthetic double-stranded RNA, poly(I:C), which is an inducer of phospho-PKR (lane 2).

A

>hg19_dna range=chr5:135415787-135416686 5'pad=400 3'pad=400 strand=- repeatMasking=none

CCACAGAACCCCTGCCTTTGGCAAGTTCATAGGCTGCTTCGGGGAGATTTTATGGAGGCCTTAGGCTGCCCCCGTCTGCTGGAGAGAACCCCGAAAAGCC
TTTGGCGGGGGTACTCCTCAGCAGCCTGG**CTGCTGGACCTAGGTAGACG**GCCCACACGCCCCCCCGCACCCTCCTGCCTCCCCTCCAGTGGGTGGATTTT
GCCCCCTTCCACGGTTCCCGCGCAGGATGCGGGCGG**GAGGAACTGAGAGTCCCTCCAGG**ACACGTTAGCAGGACGCCTGGCGCAGAGCGGGCGAGACCGC
ATGACGCAGGCCTCTCGGGGGCGGGGAGCACATGCAGCCCGTCCCTCTCCACATCGTCACTCTTCTATGGTTTAGAAGTTTCAGTCGCACACTCCTACCC
GGGTCGGAGTTAGCTCAAGCGGTTACCTCCTCATGCCGGACTTTCTATCTGTCCATCTCTGTGCTGGGGTTCGAGACCCGCGGGTGCTTACTGACCCTTT
TATGCAATAAATTC**GGTATAATCTGTCACTCTGAAGG**CTTTGTTATTTTTTATCCCTTTTAACTTTGCTAAATTAATAGGCTGATAACATAAGTTGGTGT
CACTTTGAAGGTGTGACAGAAAGTATGGAGGCTGGCCACTGGTCTTACAGGGCGTGCGTGACCAGCCTGTACTGCCAGGTAGGACGCATGGGGACAAAAA
AATGCTTGTGCAGTTCACGAGACACACCTGGAAGCTCCGAGACCCCACAACTTCGGAC**CGCGGAGTGCGTTATGGATT**TCGTCCCAAGTAGGACGGAAGT
TTAACAGCAATAGCTGCCGTCTACTGTTTTATCTGTATGACTGTTTTTTTCACATATATTGCCACCCGCCAGGACTTGCCTGTTGGACCACAGCGAGTGA

B

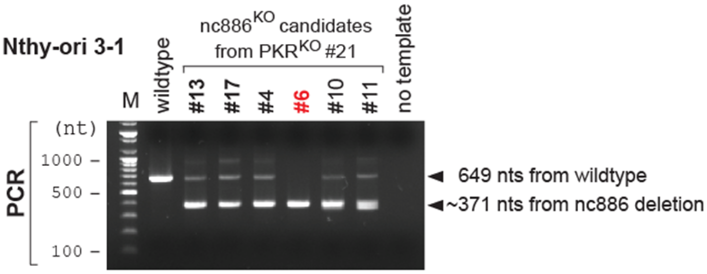

C

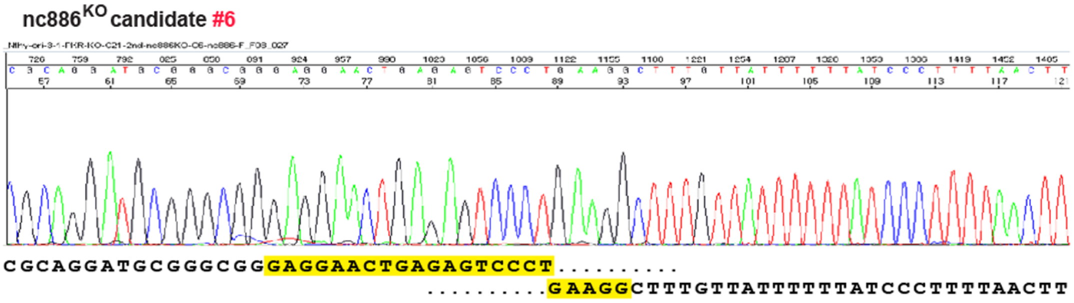

Supplementary Figure S5: CRISPR/Cas KO of nc886. A. A genomic region containing the nc886 transcript (in blue letters) and the flanking sequences. sgRNA targeting sequences are yellow-highlighted. Underlined sequences are PCR primers used in panel B. **B.** PCR amplification of the nc886 genomic sequence in nc886^{KO} candidate clones. M, molecular size markers; wildtype, genomic DNA isolated from wild type Nthy-ori 3-1 as a PCR template; no template, no template DNA added in the PCR reaction. **C.** Alignment of actual sequencing data to the reference genome sequence. sgRNAs are yellow-highlighted, showing their truncation and ligation.

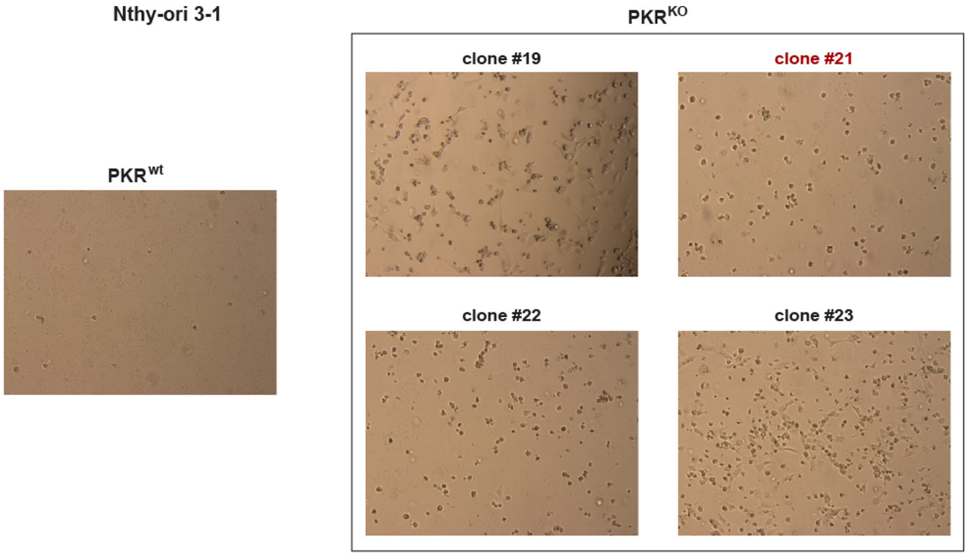

Supplementary Figure S6: Microscopic images of cells during the nc886 KO experiment. Photos were taken at three days of G418 selection after transfection of sgRNA-expressing plasmids into the indicated PKR^{wt} or PKR^{KO} Nthy-ori 3-1 cells. The clone #21 (red-highlighted) was used for further generation of $nc886^{KO}$ subclones.

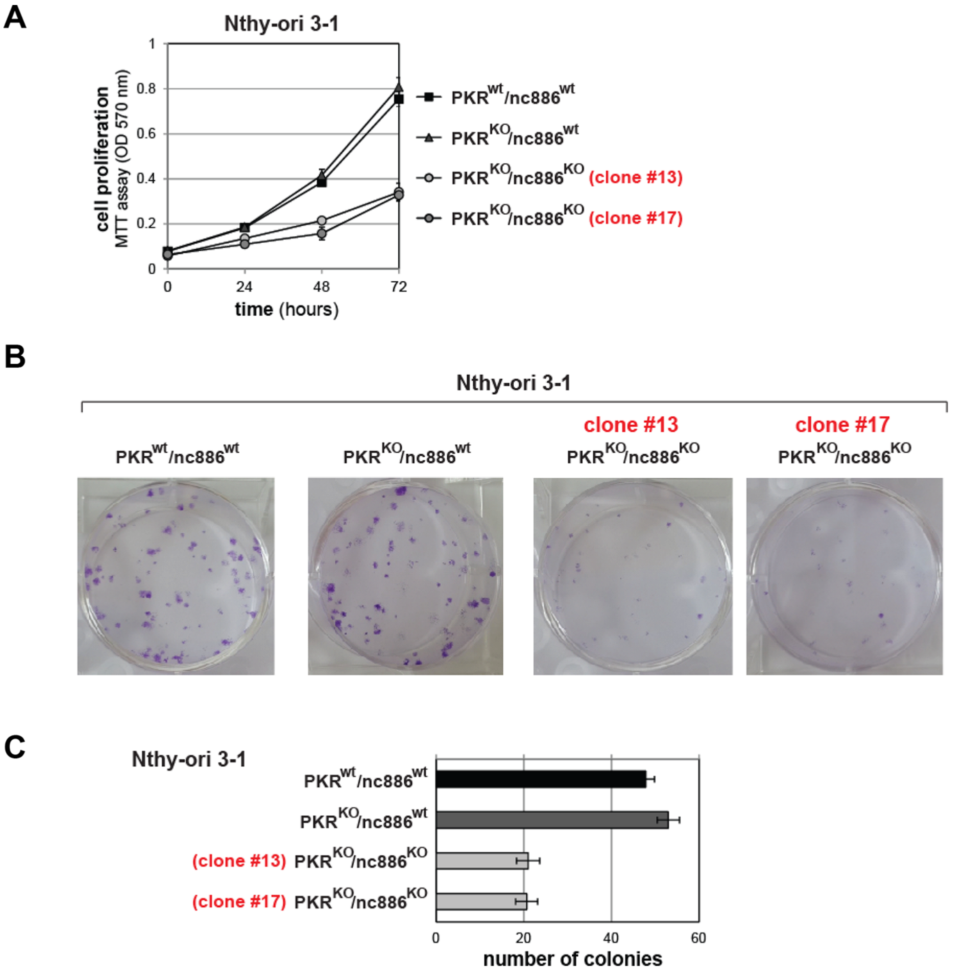

Supplementary Figure S7: Cell proliferation assays for two other nc886 KO clones. MTT cell proliferation assay **panel A.** and colony formation assays **panel B-C.** The values of PKR^{wt}/$nc886^{wt}$ and PKR^{KO}/$nc886^{wt}$ are reclaimed from Figure 3. All the other descriptions are the same as Figure 3B-D.

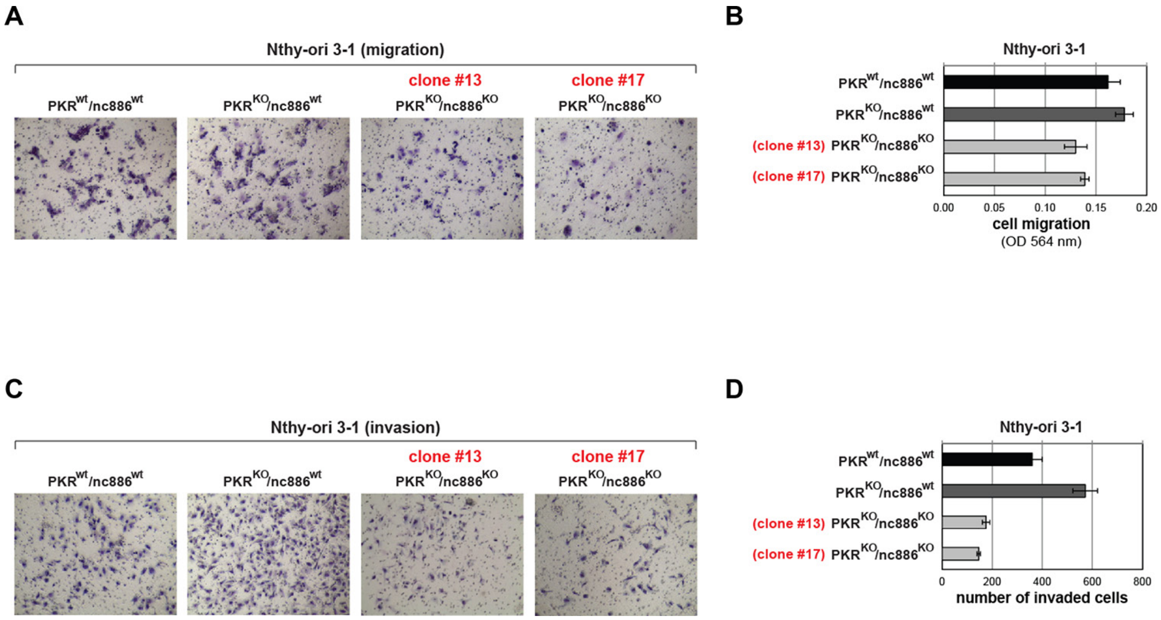

Supplementary Figure S8: Cell migration and invasion assays for two other nc886 KO clones. All the descriptions are the same as Figure 4 and S7.

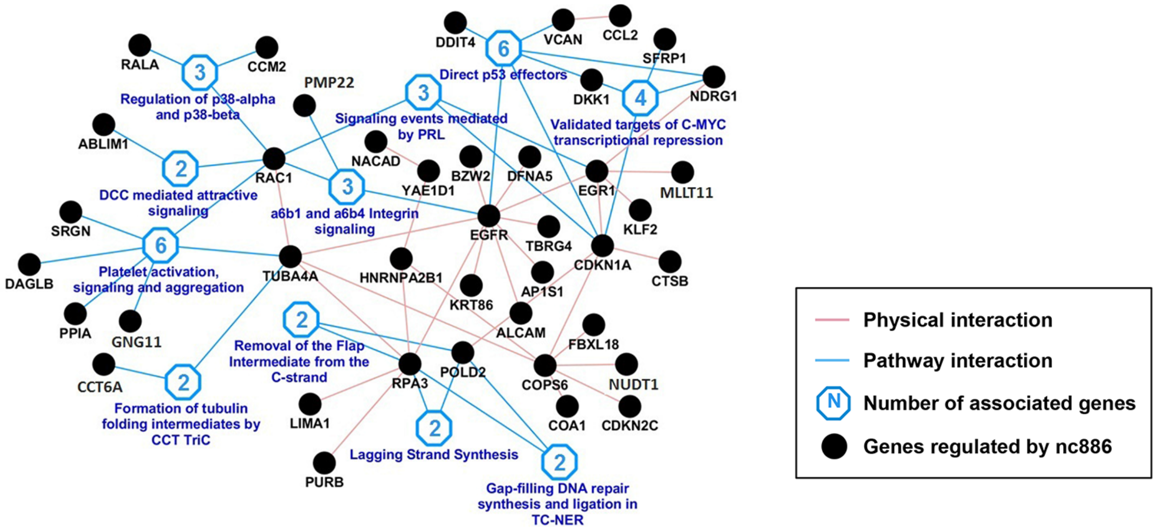

Supplementary Figure S9: Direct physical interactions and pathways of the 201 nc886-regulated genes. Red and blue edges represent direct physical and pathway interactions respectively. Blue octagons indicate pathways. For each pathway, the identity is shown below octagons and the number of associated genes is shown within octagons. Solid black circles indicate individual genes.

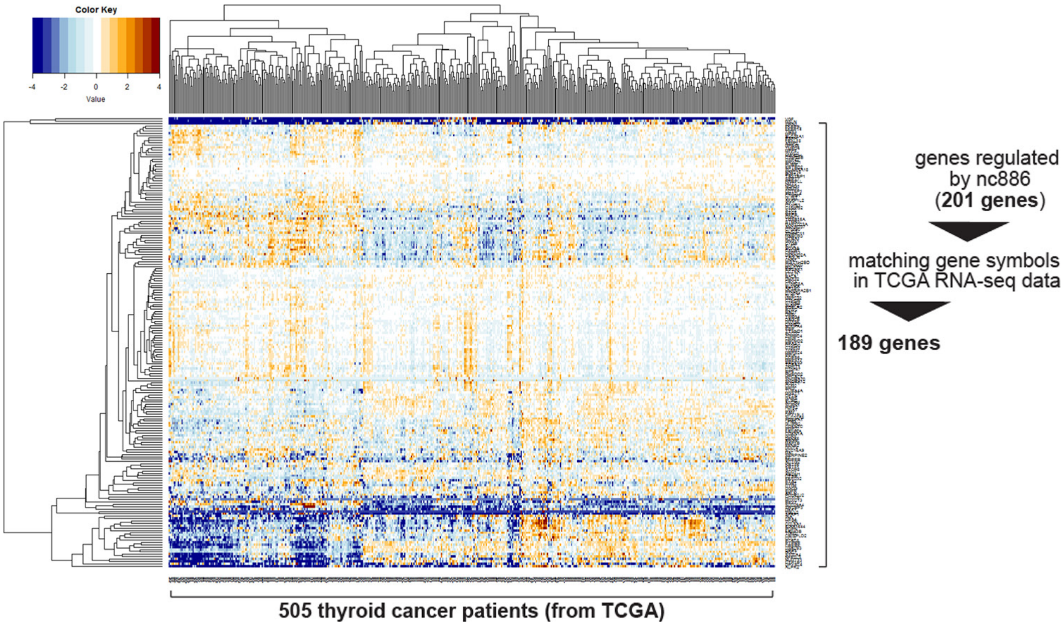

Supplementary Figure S10: The 201 nc886-regulated genes in the TCGA data. A heat map showing clustering of 201 genes (actually 189 genes; see figure captions on the right) against 505 thyroid cancer patients from the TCGA dataset.

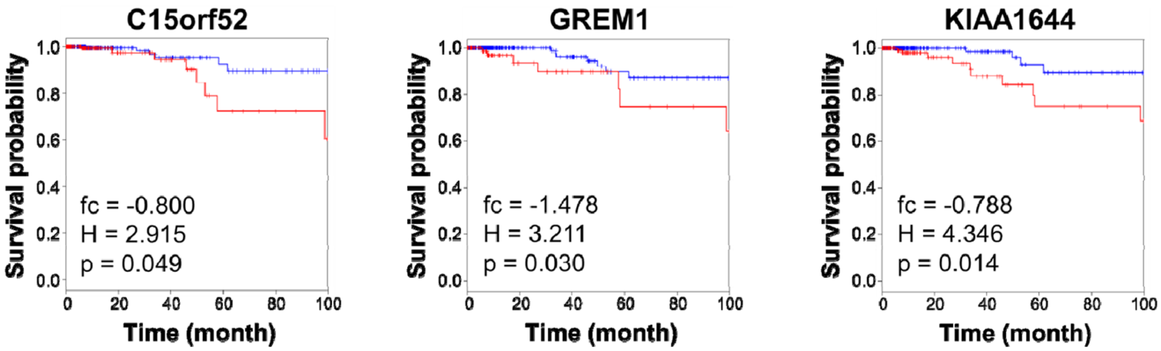

Supplementary Figure S11: Kaplan-Meier survival curves. All the descriptions are the same as Figure 5B.

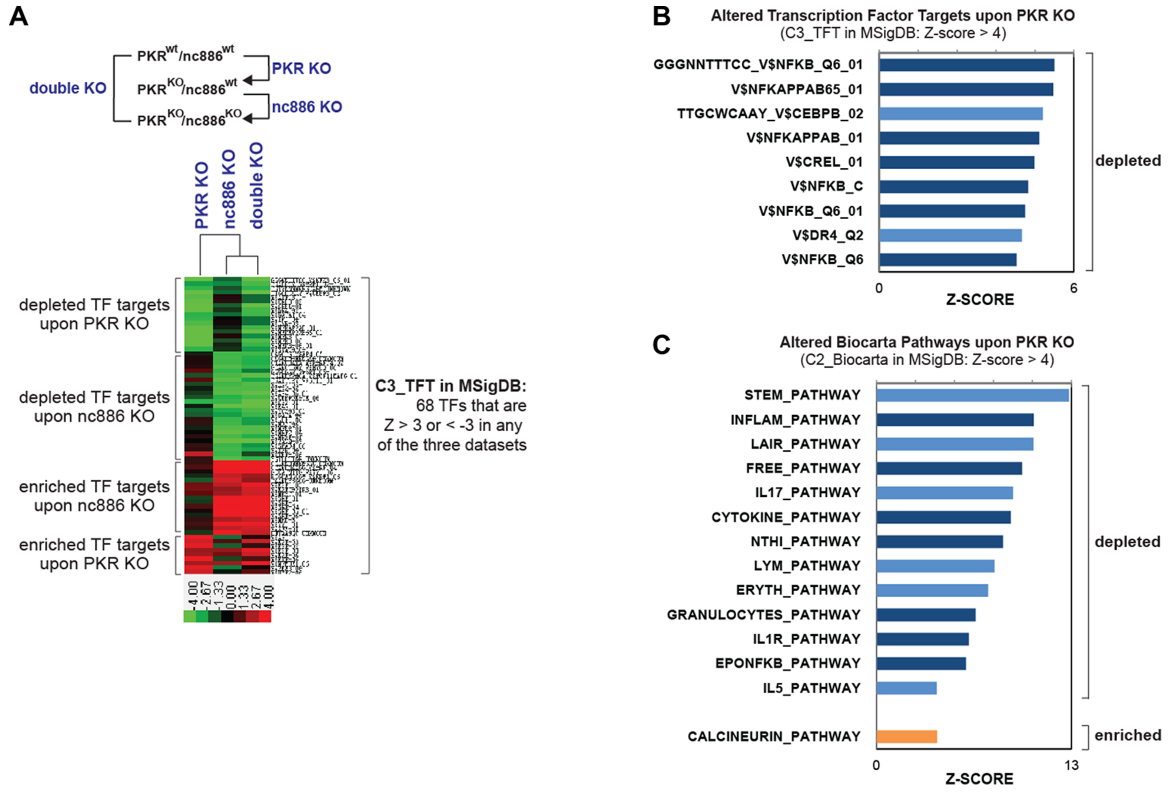

Supplementary Figure S12: NF-κB signature is downregulated upon PKR KO. A. A heat map drawn with Z-scores of 68 TF target (TFT) gene signatures (C3_TFT from Molecular Signatures Database v5.1; http://software.broadinstitute.org/gsea/msigdb/). Using Z-score cutoff = 3, 68 TFs were selected from a total of 615 TFs, whose Z-scores are fully listed in Table S2. **B.** The most enriched TFTs (Z-SCORE cutoff = 4) in PKR KO (PKR^{KO}/nc886^{wt} relative to the parental wild type Nthy-ori 3-1 cells). Dark blue bars designate NF-κB TFTs. **C.** Biocarta pathway analysis (Z-SCORE cutoff = 4) from curated gene sets (C2_BIOCARTA) in Molecular Signatures Database v5.1. Dark blue bars designate pathway in which NF-κB is implicated.

Supplementary Table S1: 226 genes significantly altered upon PKR or nc886 KO

See Supplementary File 1

Supplementary Table S2: Z-scores of 615 TF targets

See Supplementary File 2
